# RDCPF: A Redundancy-Based Duty-Cycling Pipelined-Forwarding MAC for Linear Sensor Networks

**DOI:** 10.3390/s20195608

**Published:** 2020-09-30

**Authors:** Quanwei Zhang, Dazhong Li, Yue Fei, Jiakang Zhang, Yu Chen, Fei Tong

**Affiliations:** 1 School of Cyber Science and Engineering, Southeast University, Nanjing 210096, China; quanwei_zhang@seu.edu.cn (Q.Z.); dazhong_li@seu.edu.cn (D.L.); yuefei@seu.edu.cn (Y.F.); jiakang_zhang@seu.edu.cn (J.Z.); yu_chen@seu.edu.cn (Y.C.); 2 Purple Mountain Laboratories, Nanjing 211111, China

**Keywords:** linear sensor network, duty-cycling, pipelined-forwarding, MAC, redundant nodes

## Abstract

Existing duty-cycling and pipelined-forwarding (DCPF) protocols applied in battery-powered wireless sensor networks can significantly alleviate the sleep latency issue and save the energy of networks. However, when a DCPF protocol applies to a linear sensor network (LSN), it lacks the ability to handle the bottleneck issue called the energy-hole problem, which is mainly manifested due to the excessive energy consumption of nodes near the sink node. Without overcoming this issue, the lifespan of the network could be greatly reduced. To that end, this paper proposes a method of deploying redundant nodes in LSN, and a corresponding enhanced DCPF protocol called redundancy-based DCPF (RDCPF) to support the new topology of LSN. In RDCPF, the distribution of energy consumption of the whole network becomes much more even. RDCPF also brings improvements to the network in terms of network survival time, packet delivery latency, and energy efficiency, which have been shown through the extensive simulations in comparison with existing DCPF protocols.

## 1. Introduction

The Internet of Things (IoT) has been thriving in past few decades, and its effects of linking almost every object in people’s daily lives to the Internet and augmenting items with computational power have been recognized [1]. One of the most popular research foci, and a significant technology in the IoT research area, is the wireless sensor network (WSN), which can be defined as a bunch of sensor nodes cooperating together to handle particular tasks [2]. Those tasks mostly have characteristics of sensing environmental conditions using their embedded sensors, with data packets generated and forwarded to the sink node [3]. This ability of WSNs has been widely applied to monitor linear environmental conditions around nodes for gas/oil/water pipelines, railway tracks, national borders, AC power converters, and seacoast surveillance [4]. These scenarios are usually one-dimensional in fashion, meaning they have a linear structure; therefore, sensor nodes should be deployed linearly along them, resulting in a linear sensor network (LSN) [5].

The LSN has some special traits, including: (1) The linear and narrowly distributed fashion of nodes, (2) A restricted number of neighbor nodes compared to general WSNs, (3) Necessarily long-distance radio coverage because of the sight deployment method [6], and (4) Battery-powered nodes due to the remoteness from the covering scale of electricity grid [7]. As the resulting consequence of traits (1) and (2), the delivery paths of data packets are often determined with few or no alternative tracks, and trait (3) indicates that hop-by-hop data transmission is required to finally send data to the sink node located in one end of the network. Based on those two consequences, high data transmission latency becomes a serious issue in LSN design. Trait (4) brings about another challenge that is how to ensure the energy usage efficiency of the network so that the lifespan of the network can be prolonged, which is critical in terms of the difficulty in recharging or replacing the nodes.

In order to reduce the energy waste of nodes in LSN, duty-cycling MAC protocols have been widely studied and applied, thanks to the merit of allowing nodes to sleep (e.g., to switch into low-power-listening mode [8]) for energy-saving purposes. However, such MAC protocols also bring about a gross problem called the sleep latency issue, which occurs when transceivers fail to forward data during the periodical sleeping period. It will aggravate if multi-hop transmission is applied due to the accumulativeness of latency hop by hop [9]. To overcome the issue, staggering sleep–wake schedules of nodes along the forwarding track has been an effective improvement to duty-cycling MAC protocol by letting every next-hop node along the data transmission path immediately forward a datum once it is received [1,3,9]. This feature of packet delivery strategy is named pipelined forwarding, which can significantly reduce the data transmission latency via cutting off unnecessary data packet queuing time. The existing duty-cycling and pipelined-forwarding (DCPF) protocols are presently capable of solving the aforementioned two issues in LSN. However, the feature of multi-hop transmission in LSN can easily raise the energy consumption imbalance of sensor nodes in a monitoring network [10], which cannot be avoided via merely using DCPF protocol. This issue occurs because nodes located near the sink node have to consume much more energy than do the other nodes deployed in outer region due to the higher number of packets to send [11], and it has been named the energy-hole problem.

This paper proposes a novel DCPF protocol, named redundancy-based DCPF (RDCPF), to solve the energy-hole problem by deploying redundant nodes. Nowadays, sensor nodes are becoming cheaper and cheaper, benefiting from the constant and significant evolution of wireless sensor technologies. Moreover, in RDCPF, the deployed redundant nodes only need to relay data packets but do not need to sense the environment, so they are not equipped with any sensors, which are usually the most expensive parts of nodes, making them cheaper. The main contributions of this paper are summarized as follows:

- A method of deploying redundant nodes in LSN is proposed, with a corresponding RDCPF protocol designed for data collection. The proposed solution jointly considers the energy-hole issue, the energy efficiency issue, and the packet delivery latency issue facing an LSN.
- A set of algorithms are proposed in RDCPF to tackle the challenges resulting from the redundant node deployment, including how to determine the number of redundant nodes and to deploy them, and how to keep the DCPF feature to save energy and reduce latency, while addressing the energy-hole issue.
- The proposed RDCPF protocol was evaluated through extensive simulations, which indicate that it obtains much lower data transmission latency and better energy efficiency than does a state-of-the-art DCPF protocol.

To the best of our knowledge, this work is the very first one for LSN to enhance the existing DCPF protocols by deploying redundant nodes.

The rest of this paper is organized as follows. The related work is shown in Section 2. The preliminaries of designing RDCPF protocol are shown in Section 3 and the design details are shown in Section 4. Then the performance evaluation is presented in Section 5. The paper is concluded eventually in Section 6.

## 2. Related Work

In this section, the existing related work on LSN, DCPF protocols, redundant node deployment, and energy-hole problem solutions for WSN is discussed.

### 2.1. Linear Sensor Networks

Currently, lots of researchers have put their efforts into LSNs because of the widespread requirement of monitoring linear environments. For example, the authors in [1] proposed the selective-awakening MAC protocol, in which the nodes are selectively awake depending on the node density, the traffic load conditions, and the state of the buffers of the receiving nodes. Their results show that their design is energy-efficient and has low packet loss probability. Similarly, Buratti et al. [12] proposed an MAC protocol for multi-hop LSN, which reduces the impacts of hidden/exposed node problems to make the transmission flow more efficient. In [13], the authors issued a new geographic routing sensing opportunistic approach, named EasyGo. By using layer slicing and virtual sinks, the transmission success rate arises. Later, in order to meet the requirements of multi-layered structure, Sudeep et al. [14] proposed the leach-based hierarchical routing protocol for LSNs. For node assignment in LSN, the authors in [15] presented three optimization models for determining the node density under different requirements. Deng et al. [16] tried to find out how to exploit data fusion and cooperation of the deployed sensors to enhance the network coverage performance. In recent research, unmanned aerial vehicles (UAVs) have become superior alternatives to fixed sink nodes. Christelle et al. [17] proposed an efficient way to deploy UAVs for optimal target coverage. As to modeling and analyzing the performance of an LSN, the authors in [3] proposed a physical interference model to study the impact of cumulative interference on the network data collection performance.

Overall, the aforementioned work for LSN is different from this paper, which focuses on comprehensively addressing the three common issues in LSN, i.e., energy efficiency, packet delivery latency, and energy imbalance.

### 2.2. DCPF Protocols

DCPF protocols have been widely studied and applied in sensor networks in recent years. For example, Gang Lu et al. [18] proposed DMAC, an energy efficient and low latency MAC protocol for tree-based data gathering in WSNs. Du et al. [19] proposed RMAC to deliver packets over multiple hops in a pipelined fashion. Besides the above work that focuses on the MAC layer design, cross-layer pipelined-forwarding protocols for data collection have also been concerned in the research community. In [20], P-MAC was proposed so as to solve the sleep latency problem. This work was further extended in [9,21,22]. By dividing the whole network into grades around the sink node and adding an effective node identification mechanism, the packet delivery latency and protocol overhead are reduced while the network scalability increases. The authors in [23] proposed PAX-MAC, a preamble ahead cross-layer MAC, which is a novel anycast protocol for low latency packet propagation in duty-cycled WSNs. In PAX-MAC, the route to the sink node is determined by using the preamble to propagate before the data packet is sent, and the message is sent after that, which greatly reduces the latency of the packets.

Overall, the current DCPF protocols mainly focus on the duty-cycle design to save energy and the pipelined-forwarding design to reduce packet delivery latency. However, when it comes to the energy-hole problem, which is especially urgent in LSN, the existing DCPF protocols have no consideration. Differently to them, this paper proposes an enhanced protocol to solve the energy-hole problem while keeping the DCPF advantage.

### 2.3. Redundant Node Deployment in WSN

The current researches regard redundant nodes in WSN as mainly having two roles—backup nodes and literally the redundant components in the network. For the former role, backup nodes are used to replace those which are close to being energy-exhausted [24]. On the other hand, for the latter role, redundant nodes are considered to be useless in networks, so multiple methods have been proposed to eliminate them. For example, the authors in [25] proposed an algorithm for redundancy elimination with coverage preserving in WSN. This kind of redundancy in a network is caused due to the fact that nodes in WSN are highly likely to be deployed randomly, leading many redundant nodes and data to lower the network performance.

Unlike the aforementioned work, this paper proposes RDCPF to take advantage of deploying redundant nodes to balance the network energy consumption.

### 2.4. Energy-Hole Problem Solutions

This bottleneck problem in WSNs has attracted plenty of attention. For example, in [26], the authors proposed an energy efficient routing structure using a multi-layer network model. By considering the one-to-one connection for all the gateways, the overall load on the network is balanced. In [27], the authors adopted an approach by dividing the whole network into equiangular wedges and merging them when some nodes nearly ran out of energy to provide a higher network lifetime and better energy efficiency. The authors in [28] optimized an existing routing mechanism and designed a dynamic clustering algorithm for data collection to avoid premature death of the networks. In [29], a compressive in-network data processing scheme is proposed to resolve the energy-hole problem.

Since the energy-hole problem occurs in the nodes near the sink, a mobile sink can be an intuitive solution for achieving energy consumption evenness. The authors in [30] proposed a queen honeybee migration (QHBM) algorithm to mimic queen honeybee’s behavior for the movement of the sink node. In [31], the authors proposed a inter-cluster movement algorithm for the mobile sink node to significantly balance the energy usage of cluster heads. The algorithm is based on the value of energy consumption ratio to determine the mobile sink’s location. These repositioning methods are able to increase the lifespan of the network. However, they are all of high complexity to implement due to the temporal changes. In addition, frequently reporting the current position of sink can bring about a severe overhead.

In summary, existing research on addressing the energy-hole problem is not specifically designed for the LSN with the DCPF feature, so it cannot be applied directly.

## 3. Preliminaries

RDCPF is particularly designed for the LSN with redundant nodes being deployed to solve the energy-hole problem while keeping the DCPF’s advantages. A sink node is placed at one end of the entire LSN, functioning as a gathering center of sensed data. The remaining nodes are classified into multiple grades according to their hop distances to the sink node, which is similar to the existing DCPF protocols. Naturally, the grade of the sink node is zero, the nodes one hop away from the sink node are set as grade 1, those two hops away from the sink node are in grade 2, and so on. Each data packet generated by the node with sensing ability will be delivered to its corresponding one-grade-lower node, and then forwarded hop by hop until received by the sink node.

**Figure 1:**
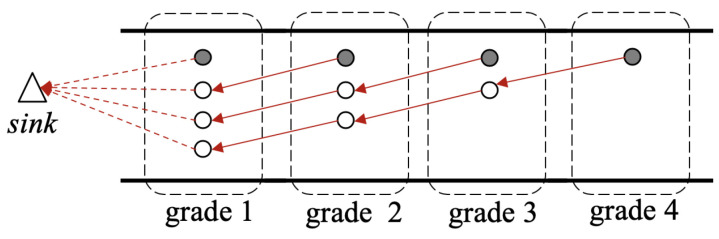
The topology of the network with a total of 4 grades. Note that each red solid-line arrow indicates the packet transmitting route of a non-grade-one node recorded in its routing table, and each red dotted-line arrow indicates the route of a grade-one node.

As mentioned in Section 1, each grade has only one sensor-equipped node (called the sensor node in this paper) to sense the ambient environment. The other nodes in the same grade without any sensors are called redundant nodes. Sensor nodes are responsible for sensing and collecting different types of information, while redundant nodes are just for relaying data packets. Considering the linear topology of the network, it is reasonable to assume that any two nodes respectively in two adjacent grades can communicate with each other. To balance the network energy consumption among all nodes so as to eliminate the energy-hole problem, the redundant nodes are deployed in such a way as that shown in Figure 1, so that each redundant node has a unique sender. Therefore, the node number in grade *i*, where i>1 and i∈N, can be determined by the following formula:

(1) $$N_{i}=N-i+1\;,$$

where *N* is the largest grade number in the network, and $N_{i}$ is the number of nodes in grade *i*. For example, N=4, as shown in Figure 1, in which each red solid-line arrow indicates the packet transmitting route of a non-grade-one node recorded in its routing table, and each red dotted-line arrow indicates the route of a grade-one node. The filled circles indicate the sensor nodes and the unfilled ones are the redundant nodes. Since a redundant node can help to relay some of the data packets received from higher-grade nodes, the energy consumption in a grade can be balanced.

**Figure 2:**
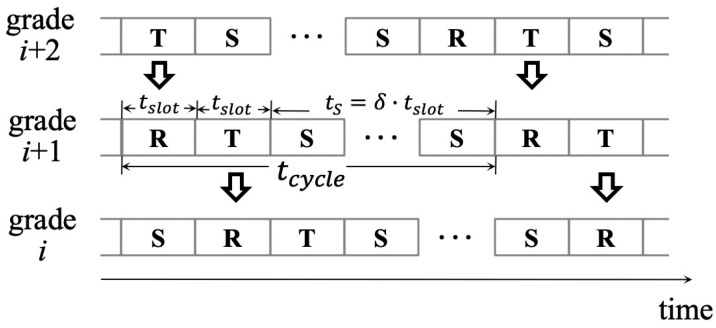
Illustration for pipelined scheduling along a packet forwarding path from grade (i+2) (i∈Nandi>1) to grade *i*.

Staggered sleep–wake schedules between each two adjacent grades are applied so that the packet delivery latency will be significantly reduced, and data packets can be forwarded along a packet forwarding path in the form of a pipeline. Specifically speaking, there are four possible states for each node in total, which are data transmission state (denoted by **T**), data reception state (denoted by **R**), sleeping state (denoted by **S**), and idle state (denoted by **Idle**). Every node is initiated to state **Idle** to listen to a channel for any possible broadcast. The sink node always keeps its transceiver on so that it can collect data all the time. Since the sleep–wake schedules are ensured between any two adjacent grades to be staggered, the packet will be forwarded immediately once it is received, as long as the grade-(i+1) node in **R** state receives a data packet from its sender in grade (i+2), and wins the contention with other potential the-same-grade forwarders before forwarding data to the next hop. Figure 2 shows an example for illustrating the DCPF working mode. It is easy to get that **R** and **T** have the same duration, known as a slot in this paper and denoted as $t_{slot}$. After state **T**, each node enters state **S** for δ (δ is a positive integer) slots. Thus, the sleep duration in a cycle is $t_{S}=\delta \cdot t_{slot}$, and one cycle duration will be $t_{cycle}=\left(\delta +2\right)\cdot t_{slot}$.

## 4. RDCPF Design

### 4.1. Problem Statements

The DCPF protocol applying to an LSN lacks the ability to handle the bottleneck issue called the energy-hole problem. Due to the characteristics of multi-hop transmission in LSN, the energy consumption of sensor nodes is unbalanced. The cause of the phenomenon is that those nodes located near the sink node have to consume much more energy than do the other nodes deployed in outer region due to the higher number of packets to send. In short, this issue is mainly caused by excessive energy consumption of nodes near the sink node. As a result, this tough issue makes the lifespan of the network greatly reduced.

**Figure 3:**
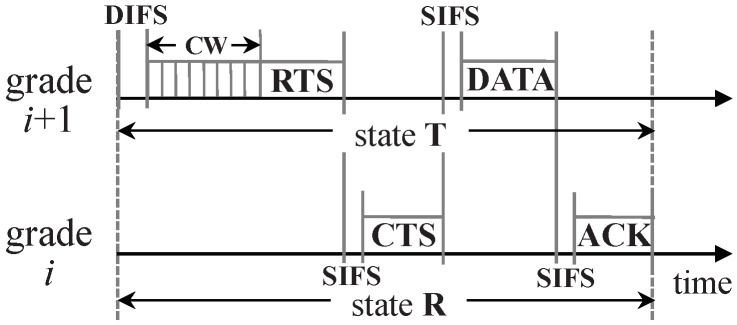
Detailed components of one slot duration.

Redundant nodes in a certain grade may have to contend for the channel to send their data packets so as to avoid collision, since they tend to be located in the interference range of each other. Thus, before two nodes process a request-to-send (RTS)/clear-to-send (CTS) handshake, a contention window (CW, similar to the backoff time in [1]) is needed for contending nodes to monitor channel for potential ongoing transmission. Figure 3 manifests the detailed components of one slot when a node in grade i+1 successfully sent a data packet to a node in grade *i*. According to Figure 3, $t_{slot}$ is computed as follows:

(2) $$t_{slot}=t_{DIFS}+t_{CW}+t_{RTS}+3\cdot t_{SIFS}+t_{CTS}+t_{DATA}+t_{ACK}\;,$$

where $t_{DIFS}$ and $t_{SIFS}$ are durations of distributed inter-frame space (DIFS) and short interframe space (SIFS), respectively; $t_{CW}$ is the duration of CW; and $t_{RTS}$, $t_{CTS}$, $t_{DATA}$, and $t_{ACK}$ are the durations needed for RTS, CTS, DATA (a data packet generated by a sensor node), and ACK (an acknowledgement packet sent by the node which received a DATA packet) transmissions, respectively.

Overall, RDCPF contains two working phases, including network initialization and data transmission. In the initialization phase, two processes are included, which are grade division and schedule establishment. In the data transmission phase, the next-hop selection process is involved if a node is transmitting a packet for the first time. This section first presents the design details of these two phases. Then an optimization method of the network topology is proposed.

### 4.2. Initialization Phase

In RDCPF, a node maintains three attributes as follows:

- G: the grade of the node, which is initialized to −1;
- S: the current state of the node, as S∈ {**R**, **T**, **S**, **Idle**}.
- T: the length of time for which the node has been in current state.

In the initialization phase, the sink node broadcasts an INITmessage in its current state (denoted as $S_{sent}$, $S_{sent}\in \left\{R,S,T\right\}$) for grade division and schedule establishment. The fields of the INIT message are shown in Table 1. Therefore, INIT.grade=0 and $\mathrm{INIT}.state=S_{sent}$. After a node receives the INIT message sent by the sink node, it starts to parse this message to determine its grade and establish the sleep–wake schedule accordingly. Then only the sensor node will rebroadcast this INIT message in its current state $S_{sent}$. Thus, INIT.grade=1, $\mathrm{INIT}.state=S_{sent}$, and the other fields are set accordingly before the message is rebroadcast.

**Figure 4:**
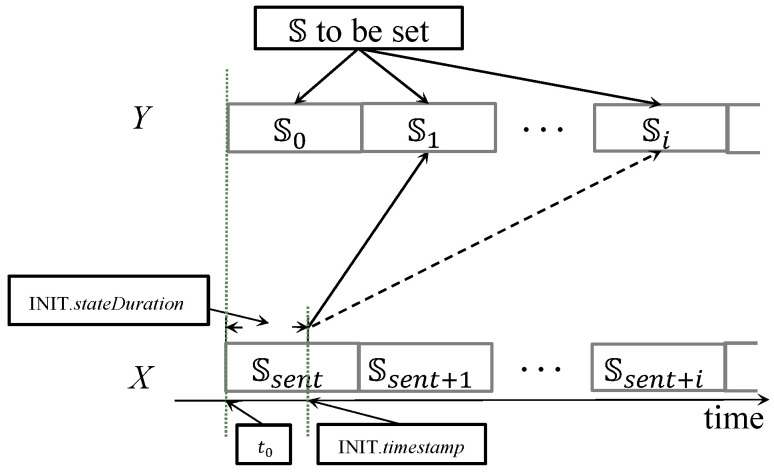
The transmission of INIT message. $S_{i}$ indicates the next $i^{th}$ state after $S_{0}$, and $S_{sent+i}$ denotes the $i^{th}$ state after $S_{sent}$.

Generally, once a node which has not joined the network receives the INIT message broadcast by a sensor node in grade (n−1), it sets its grade G=INIT.grade+1 and calculates $t_{0}=INIT.timestamp-INIT.stateDuration$, which indicates the start time of state $S_{sent}$ when the grade-(n-1) sensor node sends this INIT message in $S_{sent}$. Figure 4 shows an example for illustration, where node *X* is sending an INIT message to node *Y*. Notice that node *X* is the sensor node. The state in which node *Y* should have entered (denoted by $S_{0}$) is supposed to be staggered from the state of node *X* in $t_{0}$ (i.e., INIT.state). Therefore, $S_{0}$ can be determined as follows:

(3) $$S_{0}=\left\{\begin{array}{cc} R & INIT.state=\delta^{th}\;S\\ T & INIT.state=R\\ 1^{st}\;S & INIT.state=T\\ {\left(i+1\right)}^{th}\;S & INIT.state=i^{th}\;S\;\left(1\leq i<\delta \right) \end{array}\right.\;,$$

where $1^{st}\;S$ and ${\left(i+1\right)}^{th}$ **S** mean the first and ${\left(i+1\right)}^{th}$ slots of state **S**, as **S** contains totally δ slots.

Therefore, node *Y* can easily deduce its current state (denoted as $S_{sent}$ as aforementioned) when it is about to rebroadcast the INIT message. Node *Y* updates INIT fields, including changing INIT.grade to G of its own, INIT.state to $S_{sent}$, INIT.stateDuration to T of its own, INIT.source to its address, and INIT.timestamp to the timestamp when sending this message. Eventually, it rebroadcasts this message.

### 4.3. Data Transmission Phase

A node which has successfully established its sleep–wake schedule should start the next-hop node selection process. Beside the three attributes mentioned in the initialization phase, all nodes are required to maintain another three attributes to enable the data packet transmission process:

- FID (former ID): ID of the former node in the data transmission link (reverse of the direction wherein INIT message pass) for the current node. Should be initialized to 0;
- CID (current ID): ID of current node;
- NID (next ID): ID of the next-hop node in the data transmission link, which should be initialized to 0.

An ID is considered to be the intrinsic property of certain node which can be used to distinguish between different nodes. An ID can be the serial number set at the factory or an address in the network.

Assume **T**-state node α in grade *n* is going to send data packet to **R**-state node β (a redundant node in grade n−1). Node α applies the RTS/CTS handshake mechanism to choose one grade-(n−1) node which wins the contention as the next-hop node (i.e., β). The general message format is represented in Table 2. According to the received CTS, node α sets its NID to the source field of the message (i.e., β’s CID). Then α sends a data packet to β with its destination field set to NID. According to the received packet, β sets its FID to the source field of the packet (i.e., α’s CID). Finally, a solid routing information is maintained by both α and β.

### 4.4. Topology Optimization

**Figure 5:**
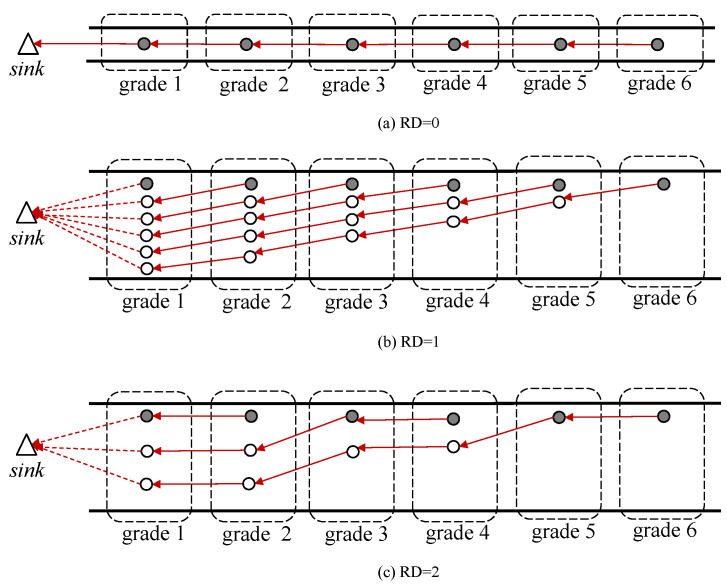
Topologies corresponding to different RD values, where total number of grades *N* is 6. Note that each red arrow indicates the packet transmitting route of a node recorded in its routing table.

According to (1), the aforementioned method of adding redundant nodes can be extremely challenging if the number of grades in the network becomes larger, since more redundant nodes are required. Thus, an enhanced method is proposed for optimizing the topology to mitigate the problem. The redundancy degree (RD) is introduced, which indicates the level of redundancy of a certain network. The higher the RD value is, the less redundant nodes the network will have. RD=0 denotes the scenario where the redundancy strategy is not used, so the proposed RDCPF protocol is degraded to a regular DCPF protocol; the aforementioned method of adding redundant nodes refers to the condition when RD=1. Intuitively, the value of RD is equal to the number of consecutive grades which contain the same number of redundant nodes. An example is shown in Figure 5 for illustration. Then the number of nodes in grade *i* is

(4) $$N_{i}=\left\lceil \frac{N-i+1}{\mathrm{RD}}\right\rceil .$$

In (4), if *i* is set to 1 and *N* respectively, the number of nodes in lowest grade (i.e., grade 1) and highest grade (i.e., grade *N*) can be obtained as $\lceil \frac{N}{\mathrm{RD}}\rceil$ and 1, respectively. Since the physical meaning of RD is defined as the number of consecutive grades which contain the same number of redundant nodes, then it is reasonable to regard those grades which have the same number of nodes as one counting unit. Therefore, there will be $\frac{N}{\mathrm{RD}}$ units containing different numbers of nodes from each other. The number of nodes in each adjacent grade has the relationship of increasing by RD from the highest grade to the lowest. Thus, by considering one counting unit as an item in arithmetic sequence, it is easy to obtain the total number of the nodes in the whole network as follows:

(5) $$N_{total}=\sum_{i=1}^{N}N_{i}=\frac{N}{2}\left(1+\left\lceil \frac{N}{\mathrm{RD}}\right\rceil \right),\;\mathrm{RD}\neq 0\;.$$

To tackle the network topology change after introducing RD, the originally proposed RDCPF should be modified accordingly. Firstly, after the grade division process in the initialization phase, all sensor nodes in their grades are supposed to know the CID information of their one-lower-grade sensor nodes. An example is shown in Figure 5 for illustration. When RD is set to 2, the sensor node in grade 4 should communicate with the sensor node instead of a redundant node in grade 3. Therefore, the CID of the grade-3 sensor node should be known by the grade-4 sensor node. To achieve this, all sensor nodes in their grades will be classified into relayable or non-relayable nodes, which indicates whether a sensor node can relay data packets for others or not. Let $SN_{i}$ denote the sensor node in grade *i*. If $SN_{i}.G\%\mathrm{RD}>0$, then $SN_{i}$ is relayable; otherwise, it is non-relayable.

After a sensor node parses the INIT message and knows its grade, it is required to judge whether it is relayable or not according to the aforementioned method. If its one-less-grade neighbor is non-relayable, then storing the CID of this neighbor is unnecessary; otherwise, the CID should be stored, because in the data transmission phase, its data packet should be delivered to its one-lower-grade relayable sensor node.

## 5. Performance Evaluation

In this section, the proposed RDCPF protocol is evaluated through the extensive OMNET++ simulations. The rest of this section consists of following four parts: the introduction to the energy consumption model, basic evaluation metrics, simulation results of the basic metrics, and a comprehensive evaluation of the network performance.

### 5.1. Energy Consumption Model

A typical energy consumption model for WSNs [10] is used as follows:

(6) $$E_{T}=\left\{\begin{array}{cc} k\cdot E_{elec}+k\cdot \epsilon_{fs}\cdot d^{2} & d<d_{0}\\ k\cdot E_{elec}+k\cdot \epsilon_{mp}\cdot d^{4} & d\geq d_{0} \end{array}\right.\;,$$

where $E_{T}$ indicates the energy consumption of transmitting *k* bits data, $E_{elec}$ is the energy consumption of transmitting circuit, $\epsilon_{fs}$ and $\epsilon_{mp}$ are required energy for power amplification under free space channel model and multipath channel model, respectively, and $d_{0}$ is a constant number with a value of 87 m. In effect, $E_{T}$ consists of two factors, including transmitting circuit loss and power amplification loss. These two factors are divided via addition operation in the equations. In the power amplification loss computation, a free space model (where $d<d_{0}$) and a multipath fading model (where $d\geq d_{0}$) are applied based on the distance between transmitter and receiver (denoted as *d*). In addition, a node receiving *k* bits data will consume $E_{R}$ amount of energy, which can be calculated as:

(7) $$E_{R}=k\cdot E_{elec}\;.$$

Note that the energy consumption of a sensor node is different from that of a redundant node. For example, the sensor module of a sensor node also consumes energy for sensing environment. The proposed scheme in this manuscript is mainly aimed at the energy consumption of LSNs in the process of transmitting messages, so the energy consumption of nodes in addition to the energy consumption of transmitting messages is not considered in the scheme. Such a consideration will not affect the evaluation of the proposed scheme itself. In addition, the model does not consider the energy consumption in the initialization process of the network.

### 5.2. Basic Evaluation Metrics

All sensor nodes in the network are assumed to be able to generate data packets conforming to a Poisson process independently with a rate of λ (packets per second). Five evaluation metrics of interest for simulation were chosen:

- Throughput: the amount of the data packets successfully received by the sink node per second.
- Average energy consumption (AEC): the average amount of energy consumption of the nodes in a grade.
- Average packet energy consumption (APEC): the average amount of energy consumption of the whole network for each node.
- Packet delivery latency (PDL): the average time that is taken for each data packet to be delivered from the sources in the same grade to the sink node.
- Network survival time (NST): the lifespan of the network, which is defined the time when the first node runs out of energy.

Among these five basic evaluation metrics, AEC and PDL can be expanded, leading to another two metrics:

- Variance of AEC: The variance value of AEC, which indicates the degree of dispersion of AEC value distribution. It can directly show the load balancing situation under the given network parameter settings. The smaller this value is, the less significant the energy hole problem remains.
- Variance of PDL: The variance value of PDL, which indicates the evenness of packet latency distribution across different grades under the given network parameter settings. The higher this value is, the less end-to-end network latency will be.

### 5.3. Simulation Results of Basic Metrics

In the following simulations, the largest grade number in the network was set to N=12. The distance between adjacent nodes was 200 m. The length of the data packet was set to 8800 bits. Note that in the proposed RDCPF protocol, when RD=0, it is degraded to a DCPF designed based on the work in [9]. Unless otherwise stated, the length of the first-in-first-out queue was set to K=3 packets.

#### 5.3.1. Throughput Versus λ

**Figure 6:**
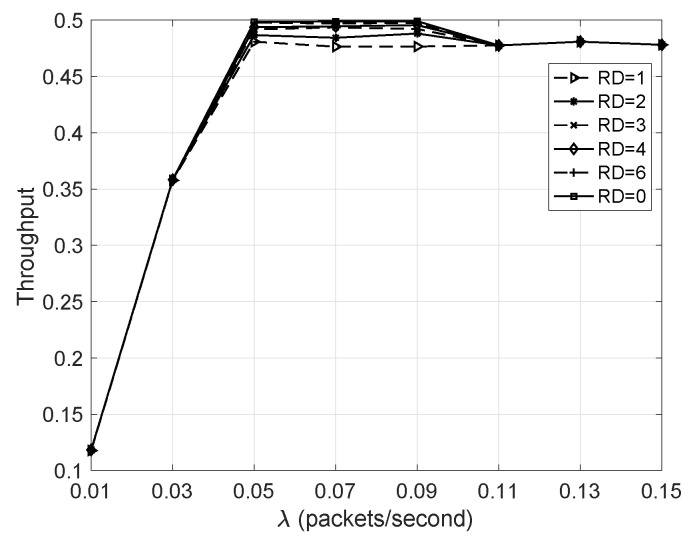
Throughput versus λ.

The redundancy-based network performance is compared with that without adding any redundant nodes. These two situations correspond to RD≠0 and RD=0 respectively. According to Figure 6, one can find that the two situations almost have the same throughput when λ≤0.05. This is because the packet arrival rate has not reached the network service rate. Therefore, for the networks with RD≠0, those nodes in the same grade do not need to contend with each other for data transmission. On the other hand, when λ>0.05, the network becomes saturated; i.e., every node always has data to send in every cycle, so the network throughput becomes almost unchanged. Since those nodes in the same grade always have to contend with each other for data transmission for the networks with RD≠0, the network throughput is a little lower than that of the network with RD=0. In the following simulations, except for the NST versus λ, λ is set to 0.07 packet/second, so that the network is saturated for each situation.

#### 5.3.2. NST Versus λ

**Figure 7:**
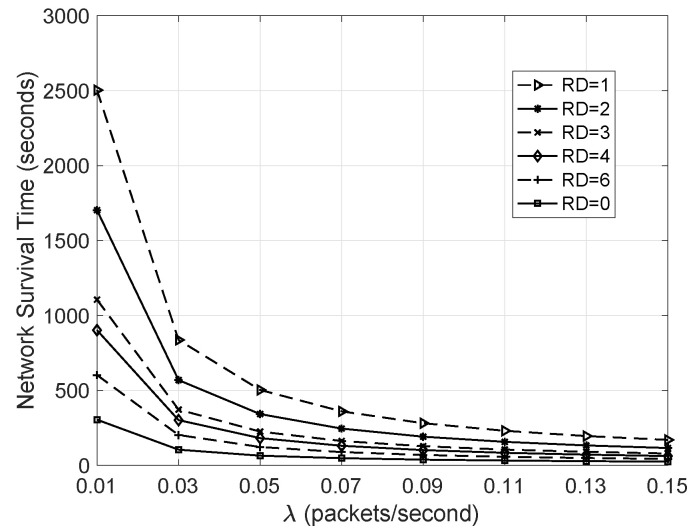
Network survival time versus λ.

Each node in the network was set to have only 1 mAh of power and kept running until there was one node running out of battery. The NST was recorded with corresponding λ, and the final result is shown in Figure 7. Obviously, when λ=0.01, the NST of the network with redundant nodes is much longer than that of the network without redundant nodes. However, as λ increases, the NST of the gap of the two networks gradually narrows. This is because when λ increases, the network with redundant nodes has serious competition during the message transmission process, resulting in greater power consumption. However, it can be seen from the figure that in this case, the NST is still longer than the NST of the network without redundant nodes. In addition, the figure also shows that as RD decreases, the network lifespan increases, since more redundant nodes are added into the network.

#### 5.3.3. AEC Versus Grade

**Figure 8:**
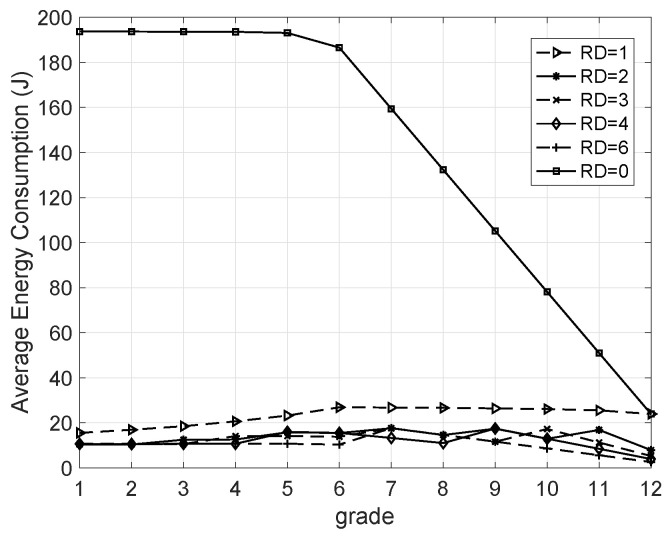
Average energy consumption versus grade.

The operating voltage of each node was set to 5 V. In addition, for the aforementioned energy consumption model shown in (6), the related parameters were set as follows: $\epsilon_{fs}=10\;pJ/bit/m^{2}$, $\epsilon_{mp}=0.0013\;\;pJ/bit/m^{4}$, and $E_{elec}=50\;nJ/bit$. After running the network for two hours, the AECs of the nodes in the same grade were collected and are shown in Figure 8. One can find that the distribution of energy consumption is very even when redundant nodes are added, meaning the energy-hole problem in LSN can be solved using the proposed protocol.

#### 5.3.4. PDL Versus Grade

**Figure 9:**
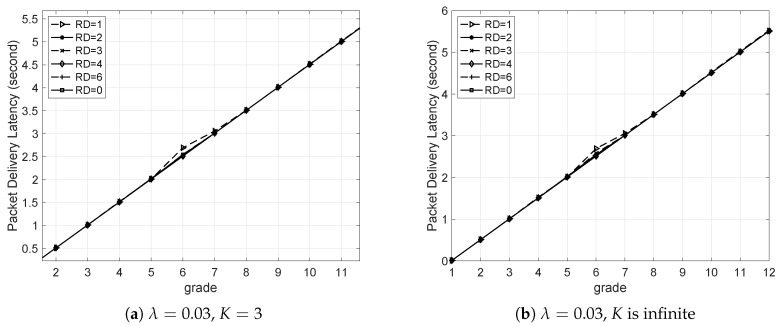

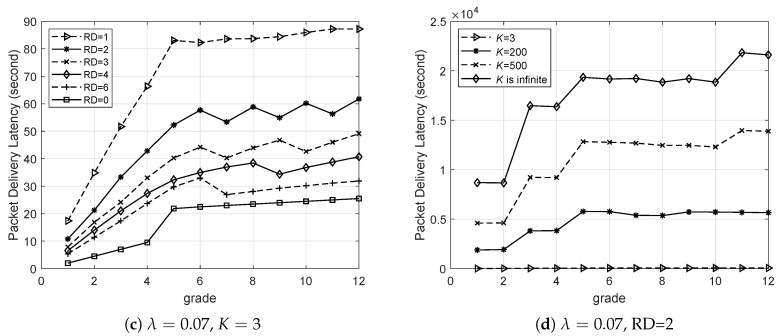
Packet delivery latency versus grade with different packet arrival rates (λ) and queue lengths (*K*).

The PDL versus grade is shown in Figure 9. First λ is set to 0.03, so that the network is unsaturated according to Figure 6. For both of the two cases, i.e., K=3 and *K* is infinite, the variance of RD almost has no impact on PDL, as shown in Figure 9a,b. This is due to the fact that when the network is unsaturated, there is almost no packet loss caused by queue overflow or packet collision in both two cases.

Then λ was set to 0.07, so that the network became saturated according to Figure 6. Each simulation was run 20 times. If *K* is set to 3, for the network with RD=0, a received packet waiting for a longer time is more likely to be dropped, which leads to a shorter PDL in comparison with the networks with larger RD, as shown in Figure 9c. This is because as RD increases, more redundant nodes are deployed, which means that there are more queues storing the long-waiting packets. However, if *K* is set to be larger or infinite, those packets waiting for a longer time will not be dropped in the network, leading to a larger PDL, as shown in Figure 9d for the case with RD = 2.

### 5.4. Comprehensive Evaluation of the Network Performance

In this subsection, a new metric, denoted as degree, is introduced to consider the comprehensive effects of some of the selected metrics listed in Section 5.2, so that it can support the network designers in determining the network parameter settings based on the customized requirements. The lower the value of degree, the better the comprehensive network performance will be.

Specifically, let *M* denote the total number of metrics to be taken into account and $v_{j}$(1≤j≤M) denote the value of the $j^{th}$ metric. To define degree, $v_{j}$ will be normalized using the min-max normalization method, shown as follows:

(8) $$n_{j}=\frac{v_{j}-v_{min}}{v_{max}-v_{min}}\;,$$

where $v_{min}$ and $v_{max}$ are the minimum and maximum values of all the considered metrics, respectively, and $n_{j}$ is the normalized value of the $j^{th}$ metric. Notice that a different metric may contribute positively or negatively. Then degree can be quantified as follows:

(9) $$degree=\sum_{j=1}^{M}w_{j}m_{j}\;,$$

where $m_{j}=n_{j}$, if the $j^{th}$ metric contributes positively; otherwise $m_{j}=-n_{j}$. $w_{j}$ is the weight of the $j^{th}$ metric and $\sum_{j=1}^{M}w_{j}=1$. Notice that $w_{j}$ indicates the designer’s concern about the weight of the $j^{th}$ metric. For instance, if the network designers care most about the network throughput, they can set the corresponding weight to the greatest one among all of the considered metrics.

There were M=6 metrics considered, including the AEC variance, the PDL variance, throughput, APEC, NST, and the total number of nodes. Since this paper focuses on solving the energy-hole problem, the AEC variance is the first priority among all the considered metrics, and its weight was set to 0.5. The weights of the left five metrics were all set to 0.1. The considered metrics and the corresponding parameters related to degree are listed in Table 3. The results of preprocessing data are shown in Table 4. As indicated in the footnotes of Table 4, different values were used to determine the values of $m_{1}$ to $m_{5}$ based on their respective focuses. When $m_{1}$, $m_{2}$, and $m_{4}$ were measured, *K* was set to be infinite, because the focus was on the performance estimation of the whole path through grades 1 to 12. Thus, the network operators were supposed to ensure that all packets were delivered by setting *K* to be infinite, so that those metrics could reflect the network performance more precisely. On the other hand, the metric throughput was only required to show the sink node’s ability to handle the packets sent by nodes in grade 1, making the setting of *K* being infinite unnecessary. NST was measured in the scenario where the battery capacity was set to be quite low so that the simulation results could be quickly obtained. Finally, since the min-max normalization method was applied, the absolute values of these metrics have little to do with the final result, so it is safe to say that this approach, using different scenarios to obtain the parameters in Table 4 to conclude on the best RD value, is reasonable. Then, the corresponding value of degree for each RD can be calculated. The results are shown in Table 5.

According to Table 5, it is obvious that RD=4 is the best choice. Note that this conclusion was obtained based on the intention of providing an evaluation method by comprehensively considering several evaluation metrics of interest. A specific RD may not be the best set when it is judged by each evaluation metric separately, but it may come to be the best when this scenario is judged by all metrics of interest according to a set of certain weights, as shown in Table 3.

## 6. Conclusions

This paper introduced a method of adding redundant nodes in LSN to solve the bottleneck problem, i.e., the energy-hole problem, facing the existing DCPF protocols. By proposing a novel MAC protocol, called RDCPF, the energy consumption can be evenly distributed among network nodes, since all the sensor nodes in the network do not need to relay data packets for others and each redundant node relays data packets for only one sensor node. Meanwhile, the DCPF feature is still kept to save energy and reduce packet delivery latency. The extensive simulation results manifest that the proposed solution to the load imbalance issue and the energy-hole problem outperforms the existing DCPF protocols in terms of packet delivery latency, average power consumption, network survival time, and energy usage efficiency.

In future work, the scenario where there is more than one sensor node (equipped with sensors) in each grade will be considered, since in the current work only the scenario where there is only one sensor node deployed in each grade was considered. The corresponding protocol will be designed and evaluated as well. In addition, in the current work, those nodes in the same grade need to contend with each other for data transmission, as they maintain the same sleep–wake schedule. Especially when the traffic load becomes higher, such contentions become more serious, leading to more unsuccessful data transmissions. In the future work, a scheme will be designed to eliminate this issue. Last but not least, a hardware testbed will be built in the future work to implement and evaluate the proposed protocol, so as to make the protocol more robust and realistic.

## Figures and Tables

**Table 1:** INIT message contents.

Fields	Descriptions
*grade*	The grade in which node *Y* is.
*state*	The state at which node *Y* stays when it emits INIT message.
*stateDuration*	The duration that node *Y* has resided in current state right before sending this INIT message.
*source*	The address of node *Y*.
*timestamp*	The time of the moment in which node *Y* emits INIT message.

Assume that the INIT message is sent by node *Y*.

**Table 2:** The general message format.

Fields	Descriptions
*type*	The type of this message (RTS/CTS/ACK/DATA)
*grade*	The grade where the message’s generator is
*destination*	The destination of this message. If it is set to 0, then
	any nodes receive this message are allowed to contend for response
*source*	The CID of the sender of this message
*data*	The content of sensed data (only available when type of this message is DATA)

**Table 3:** Metrics with corresponding parameters.

*j*	Metric	$m_{j}$	Weight
1	the variance of AEC	$-n_{1}$	0.5
2	the variance of PDL	$-n_{2}$	0.1
3	throughput	$+n_{3}$	0.1
4	APEC	$-n_{4}$	0.1
5	NST	$+n_{5}$	0.1
6	the total number of nodes	$-n_{6}$	0.1

**Table 4:** Results of data preprocessing.

RD	${m_{1}}^{\;1}$	${m_{2}}^{\;1}$	${m_{3}}^{\;2}$	${m_{4}}^{\;1}$	${m_{5}}^{\;3}$	$m_{6}$
1	−0.0019	−0.1032	0	−0.2129	1	−1
2	0	−0.0747	0.3540	−0.0987	0.6322	−0.4545
3	−0.0005	−0.0081	0.7640	0	0.3672	−0.2727
4	−0.0009	−0.0011	0.8075	−0.0074	0.2650	−0.1818
6	−0.0012	0	0.9193	−1.0000	0.1303	−0.0909
0	−1.0000	−1.0000	1.0000	−0.4092	0	0

${}^{1}$ Data obtained when the network operating duration is 2 h, and the packet loss is not considered with an infinite queue and λ=0.07. ${}^{2}$ Data obtained when the network operating duration is 2 h, and the packet loss is considered with K=3 and λ=0.07. ${}^{3}$ Data obtained when each node in the network contains only 1 mAh battery power with λ=0.07.

**Table 5:** Calculation results of degrees.

RD	1	2	3	4	6	0
degree	0.0326	−0.0358	−0.0848	−0.0878	0.0048	0.5409
